# The N-terminal sequence of the extrinsic PsbP protein modulates the redox potential of Cyt *b*_559_ in photosystem II

**DOI:** 10.1038/srep21490

**Published:** 2016-02-18

**Authors:** Taishi Nishimura, Ryo Nagao, Takumi Noguchi, Jon Nield, Fumihiko Sato, Kentaro Ifuku

**Affiliations:** 1 Graduate School of Biostudies, Kyoto University, Sakyo-ku, Kyoto 606–8502, Japan; 2 Graduate School of Science, Nagoya University, Aichi 464–8602, Japan; 3 School of Biological and Chemical Sciences, Queen Mary University of London, London E1 4NS, United Kingdom

## Abstract

The PsbP protein, an extrinsic subunit of photosystem II (PSII) in green plants, is known to induce a conformational change around the catalytic Mn_4_CaO_5_ cluster securing the binding of Ca^2+^ and Cl^–^ in PSII. PsbP has multiple interactions with the membrane subunits of PSII, but how these affect the structure and function of PSII requires clarification. Here, we focus on the interactions between the N-terminal residues of PsbP and the α subunit of Cytochrome (Cyt) *b*_559_ (PsbE). A key observation was that a peptide fragment formed of the first N-terminal 15 residues of PsbP, ‘pN15’, was able to convert Cyt *b*_559_ into its HP form. Interestingly, addition of pN15 to NaCl-washed PSII membranes decreased PSII’s oxygen-evolving activity, even in the presence of saturating Ca^2+^ and Cl^–^ ions. In fact, pN15 reversibly inhibited the S_1_ to S_2_ transition of the OEC in PSII. These data suggest that pN15 can modulate the redox property of Cyt *b*_559_ involved in the side-electron pathway in PSII. This potential change of Cyt *b*_559_, in the absence of the C-terminal domain of PsbP, however, would interfere with any electron donation from the Mn_4_CaO_5_ cluster, leading to the possibility that multiple interactions of PsbP, binding to PSII, have distinct roles in regulating electron transfer within PSII.

The oxygen-evolving reactions are a fundamental component of Life and critical to the evolutionary success underpinning the conversion of sunlight to chemical energy. This chemistry is performed within a protein-ligand-cofactor micro-environment termed the Oxygen-Evolving Complex (OEC), extending out from the lumenal surface of membrane-bound photosystem II (PSII)^1^. Much progress has been made toward determining the structure of the PSII complex and recent X-ray structural analysis of the prokaryotic, cyanobacterial, PSII complex at atomic resolution has revealed the location of >20 membrane-intrinsic and -extrinsic protein subunits, pigments, and redox cofactors, including a metal cluster of four Mn ions, Ca^2+^, and five oxo ligands, together termed the Mn_4_CaO_5_ cluster^2^,^3^.

Light excitation of the primary donor P680, a special pair of chlorophyll (Chl) *a* dimers in PSII, leads to primary charge separation and subsequent electron transfer to a nearby pheophytin, which is followed by further electron transfer via two quinones, Q_A_ and Q_B_. The oxidative hole remaining on P680 is transferred to the Mn_4_CaO_5_ cluster via a redox-active tyrosine, Tyr^161^, on the D1 subunit. The Mn_4_CaO_5_ cluster converts two water molecules into one molecule of oxygen and four protons through a light driven cycle consisting of five intermediates called S_*i*_ states (*i* = 0–4)^4^. Among them, the S_1_ state is the most dark-stable, and flash illumination advances each S_*i*_ state (*i* = 0–3) to the next S_*i*+*1*_ state. Molecular oxygen is released during the S_3_–S_4_–S_0_ transition after the transient S_4_ state^5^.

In addition, PSII has a side-electron pathway mediated by at least the Cyt *b*_559_, formed of the PsbE and PsbF subunits, carotenoids, and a chlorophyll, Chlz, that together function as a safety valve to remove the excess oxidative hole from the donor side, although the detail of this side-electron pathway is still a subject of much debate^6^. Cyt *b*_559_ is known to have several forms that differ in their redox potential: the high-potential (HP) form, the intermediate-potential (IP) form and the low-potential (LP) form^7^. It has been hypothesized that Cyt *b*_559_ may interconvert amongst its various redox states dependent upon any ongoing donor- and acceptor-side inhibition of PSII, and thus contributes to the protection of PSII from photodamage^8^.

The composition of membrane-intrinsic PSII core subunits is highly conserved among photo-oxygenic organisms, while the make-up of the extrinsic protein domain has undergone significant change during evolution^9^. Green plants, including higher plants, have a set of three extrinsic proteins, PsbO, PsbP, and PsbQ^10^. In contrast, cyanobacteria have PsbO in common, but feature PsbV (Cyt *c*_550_) and PsbU instead of PsbP and PsbQ^11^. It has also been reported that cyanobacteria possess PsbP and PsbQ homologs, termed CyanoP and CyanoQ^12^. The current view is that PsbV and PsbU have been lost during evolution, and PsbP and PsbQ in green plants appear to have evolved from CyanoP and CyanoQ, respectively^13^. Furthermore, higher plants have multiple homologs of PsbP and PsbQ. In Arabidopsis, two PsbP proteins (PsbP1 and PsbP2), two PsbQ proteins (PsbQ1 and PsbQ2), two PsbP-like proteins (PPL1 and PPL2), seven PsbP-domain proteins (PPD1-7), and three PsbQ-like proteins (PQL1-3) have been identified^14^. Genetic studies using Arabidopsis mutants have demonstrated that PsbP and PsbQ homologs are actually involved not only in PSII regulation and PSII repair, but also in chloroplast NDH activity and PSI assembly^15^,^16^. However, the exact reason as to why green plants have developed PsbP and PsbQ, specifically, for binding to PSII, remains to be answered^17^.

The molecular function of the PsbP and PsbQ proteins has been studied both *in vitro* and *in vivo*. For the former, *in vitro* release-reconstitution experiments using isolated oxygen-evolving PSII membranes have shown PsbP and PsbQ to be responsible for the retention of Ca^2+^ and Cl^–^ within the OEC, essential cofactors for the oxygen-evolving, or water-splitting, reactions^18^,^19^. Fourier transform infrared (FTIR) difference spectroscopy has elucidated that PsbP, but not PsbQ, induces protein conformational changes around the Mn_4_CaO_5_ cluster to modulate the binding properties of Ca^2+^ and Cl^−^ 20. Analysis of knockout and knockdown plants has revealed that PsbP is essential for plant photo-autotrophy and assembly of PSII^21^,^22^,^23^,^24^, while PsbQ is only required for PSII stability under low light conditions^25^. Therefore, the interaction between PsbP and PSII is particularly important for optimising and enhancing oxygen-evolution, while PsbQ has an auxiliary function to stabilize the functional interaction of PsbP with PSII^26^.

Recent chemical cross-linking experiments using 1-ethyl-3-(3-dimethylaminopropyl) carbodiimide (EDC) suggest that PsbP has multiple interactions in a higher plant PSII supercomplex; PsbP directly interacts with the Cyt *b*_559_ α subunit (the PsbE protein) via its N-terminus, and also with PsbR^27^. In addition, PsbP interacts with both CP26 and CP43 light-harvesting proteins via the amino-acid residues located in its C-terminal domain^28^. We previously reported that PsbP-∆15, in which the highly conserved N-terminus 15-residues are truncated, loses the ability to induce protein conformational change around the Mn_4_CaO_5_ cluster, and did not induce any oxygen-evolution^20^,^29^. This N-terminal sequence of PsbP is invisible in the current structural models and should take an extending flexible structure^30^,^31^. The binding of PsbQ, however, can restore the function of PsbP-∆15^26^, indicating that the N-terminal sequence of PsbP is not essential for the retention of ions in the OEC and may have other functions.

In this study, we investigated the importance of the interaction of PsbP with PSII via its N-terminus. Reduced-minus-oxidized spectra of Cyt *b*_559_ showed that the redox potential change of Cyt *b*_559_ occurs through association of native PsbP protein, but not by N-terminal truncated PsbP. Intriguingly, a synthetic pN15 peptide, which consists of PsbP N-terminal 15 residues, affects the structure of Cyt *b*_559_ in a transmembrane manner and triggers the redox potential change of the haem in Cyt *b*_559_. Furthermore, the pN15 peptide reduces the oxygen-evolving activity of PSII and inhibits the S_1_ to S_2_ transition in the OEC suggested by thermoluminescence (TL) and FTIR. The above observations indicate for the first time that a novel mechanism exists for modulating the redox property of Cyt *b*_559_ which may simultaneously affect the internal electron transfer within PSII by a lumenally bound extrinsic subunit, PsbP.

## Results

### N-terminal sequence of PsbP modulates the redox potential of Cyt *b*_559_ in PSII

**Figure 1:**
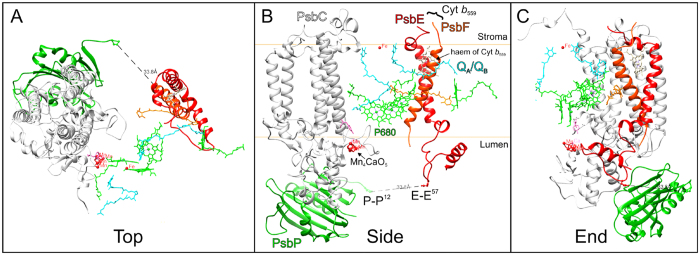
A presumptive model showing the binding of PsbP to the PSII complex. Set within an approximate membrane domain certain intrinsic and extrinsic subunits, CP43 (grey), Cyt *b*_559_ (red), and PsbP (green) are shown in cartoon ribbon form. The particular cofactor molecules, P680 (green), ChlZ (green), Q_A_ (blue), Q_B_ (blue), haem (beige), β-carotene (orange) are rendered in stick model form. The Mn_4_CaO_5_ cluster and TyrZ of the D1 subunit (D1-Tyr^161^; in pink) are also presented, but visualised as surface rendered spheres and stick models, respectively. The Pro^12^ in PsbP and Glu^57^ in PsbE, are shown as stick models, accompanied by estimated distances. UCSF Chimera^62^ was used as the software modelling environment.

Figure 1 depicts the presumptive binding model of the latest X-ray structure of spinach PsbP protein (PDB ID 4RTI)^31^ fitted into the cyanobacterial PSII structure (PDB ID 3ARC)^2^ based upon chemical cross-linking experiments^27^,^28^. Only the membrane protein subunits that would interact directly with PsbP are shown. PsbP is proposed to bind to CP43 whilst extending its N-terminal sequence to interact with PsbE on the thylakoid lumenal side^28^. Although the N-terminal 11 residues of PsbP are disordered, being absent from the crystal structure, they have sufficient length to reach any site cross-linked by EDC (Ala^1^ in PsbP and Glu^57^ in PsbE). Another model for PsbP localization has also been proposed, where the N-terminal sequence of PsbP takes on a more compact structure, but still maintains the possibility of interacting with PsbE^32^. Due to these differences, we decided to investigate more thoroughly the effect of such interactions, between the PsbP N-terminus and the PsbE subunit of Cyt *b*_559_, on the overall structure and function of PSII.

**Figure 2:**
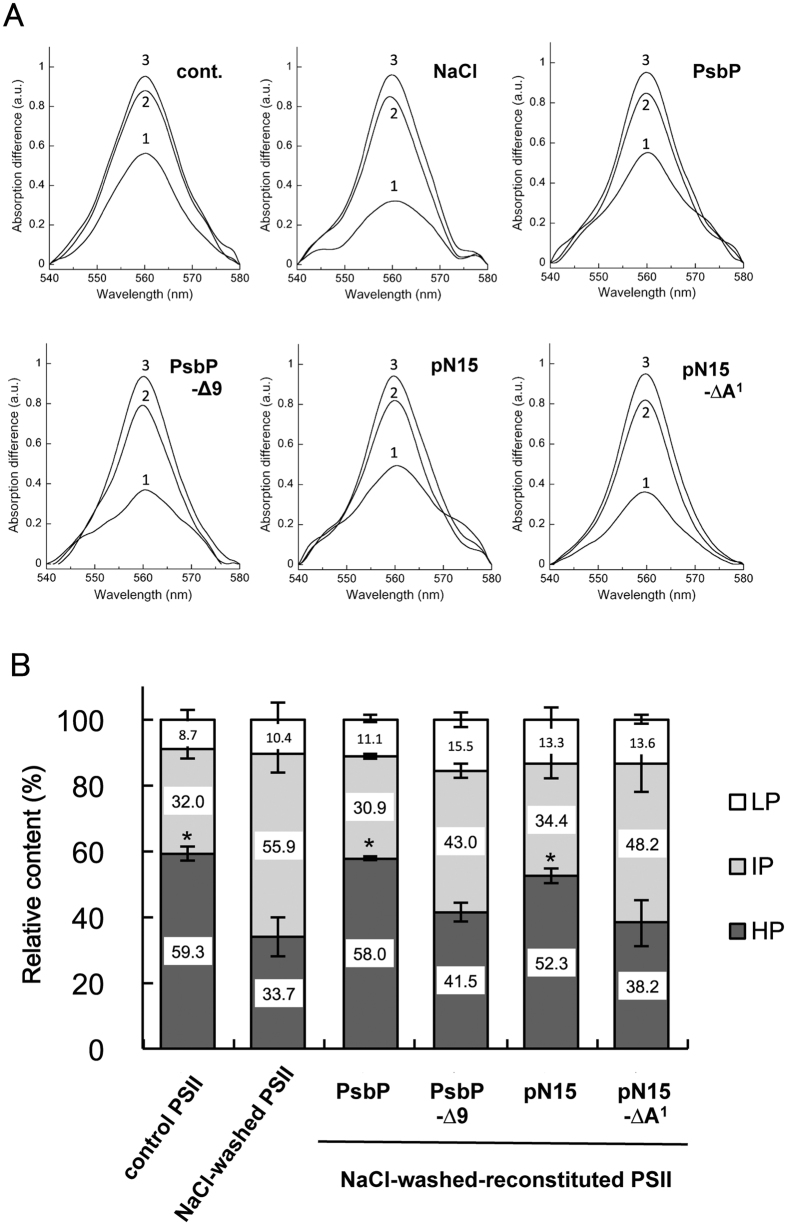
Changes in the redox potential of Cyt *b*_559_ when induced by PsbP. (**A**) Reduced minus oxidized spectra of Cyt *b*_559_ in untreated (cont.), NaCl-treated (NaCl), PsbP-reconstituted (PsbP), PsbP-∆9-reconstituted (PsbP-∆9), pN15-recontituted (pN15), and pN15-∆A^1^-reconstituted (pN15-∆A^1^) PSII membranes. Hydroquinone-reduced minus ferricyanide-oxidized (1), ascorbate-reduced minus ferricyanide-oxidized (2), and dithionite-reduced minus ferricyanide-oxidized (3) difference spectra were used to estimate the amount of the HP, IP, and LP forms of Cyt *b*_559_, respectively. Each spectrum was an average of three independent measurements and normalized to the peak of the dithionite-reduced minus ferricyanide-oxidized difference spectrum. (**B**) Relative content of the HP, IP, and LP forms of Cyt *b*_559_ determined by stepwise reductive titration. Total amount of Cyt *b*_559_ is set as 100%. The asterisks indicate the significant increase of the content of the HP Cyt *b*_559_ by reconstitution. (*p < 0.05, Student’s *t*-test); *n* = 3, error bars = SEM.

Firstly the effect of the association of native PsbP and PsbP-∆9 was investigated, the latter a mutated PsbP protein which lacks the last 9 residues of the N-terminus but retains the ability to activate the oxygen-evolving abilities of PSII^10^,^33^. Spinach PSII membranes were treated with 1.5 M NaCl to remove native PsbP and PsbQ proteins, and then the PsbP protein was reconstituted back to the NaCl-washed PSII membranes at a molar ratio of 4:1 (PsbP:PSII) (Fig. S1). All redox forms of Cyt *b*_559_ were first oxidized by ferricyanide, followed by its stepwise reduction with hydroquinone, ascorbate, and dithionite, all of which can be monitored by absorption difference spectroscopy^34^. An averaged reduced-minus-oxidized spectrum for each PSII sample is shown in Fig. 2A, and the ratios of various redox forms of Cyt *b*_559_ are presented in Fig. 2B. Intact PSII membranes were observed to contain about 59% of the HP form, 32% of the IP form, and 9% of the LP form. In NaCl-washed PSII membranes, the HP content was reduced to about 34% and instead the IP and LP contents were increased. In this way the dissociation of PsbP and PsbQ induced the conversion of Cyt *b*_559_ into its lower redox potential form, while reconstitution of PsbP restored the HP content to about 58%, as reported previously^35^,^36^. However, reconstitution of PsbP-∆9 was less efficient in restoring the HP content, indicating that the N-terminal sequence of PsbP was playing a significant role in the plant’s ability to restore HP Cyt *b*_559_ into PSII.

Surprisingly, reconstitution of pN15 alone, a peptide fragment consisting of the first N-terminal 15 residues of PsbP (NH_2_-AYGEAANVFGKPKKN-CONH_2_), at the molar ratio of 200:1 (pN15:PSII), was able to restore the amount of the HP form to ~52% without activating the OEC (Fig. S2). This effect was not observed with the peptide when it was lacking its N-terminal Ala^1^ (pN15-∆A^1^). The above results suggest that the interaction of the N-terminal sequence of PsbP with PsbE, on the lumenal side of the thylakoid, affects the redox properties of the haem of Cyt *b*_559_ in a manner that traverses the membrane and one that is independent from the oxygen-evolving activity of PSII.

### The pN15 fragment interacts with PsbE and alters the conformation of Cyt *b*_559_

**Figure 3:**
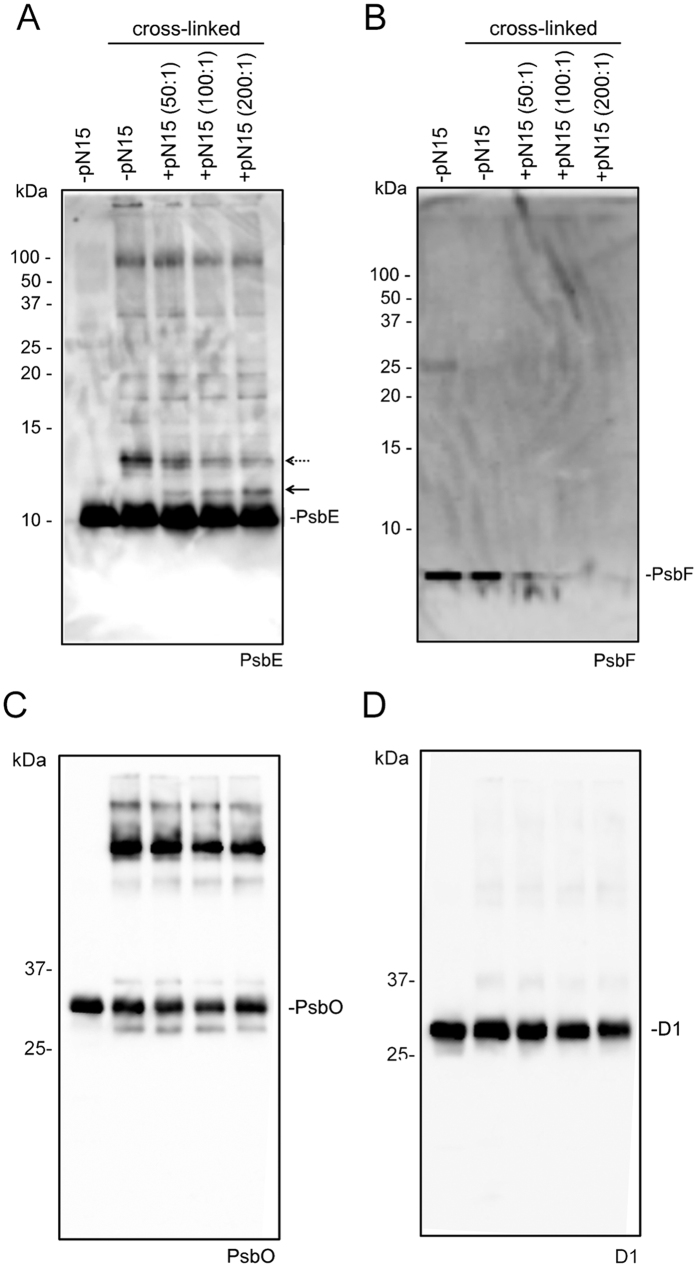
Cross-linking of the pN15 fragment with PSII membranes using EDC and sulfo-NHS. NaCl-washed PSII membranes were cross-linked with pN15 at a molar pN15:PSII ratio of 50:1 to 200:1. For each sample, an amount of protein corresponding to 3 μg Chl was loaded onto each lane and detected with antisera against PsbE (**A**), PsbF (**B**), PsbO (**C**), and D1 (**D**). The arrow at ~11 kDa indicates the peptide of pN15 cross-linked to PsbE. The dashed arrow at ~13 kDa indicates the putative peptide of PsbE cross-linked to PsbF. The original positions of PsbE (9 kDa), PsbF (4 kDa), PsbO (33 kDa), and D1 (32 kDa) subunits are also indicated.

To confirm that pN15 interacts with PsbE in PSII, pN15 was cross-linked to PSII using EDC, a chemical cross-linker. EDC, a zero length cross-linker, cross-links a primary amine and a carboxyl group when electrostatically associated. Subsequently, the cross-linked PSII complexes were analysed by SDS-PAGE and any cross-linked products were visualized by immunoblotting using specific antibodies. The results of the immunoblotting, for PSII membranes cross-linked in the presence or absence of pN15 reconstitution, are shown in Fig. 3. No specific band appeared in the PsbO and D1 immunoblots, indicating that pN15 did not affect those particular subunits (Fig. 3C,D). A specific band did appear in the PsbE immunoblot in the presence of pN15 at approximately 11 kDa (Fig. 3A). This band was not observed when PSII was treated with EDC in the absence of pN15 and its intensity was dependent upon the amount of pN15 used during reconstitution. The molecular mass of this cross-linked product was consistent with the theoretical molecular mass of the pN15-PsbE cross-linked product (MW: 10.8 kDa).

Notably, the intensity of the band at approximately 13 kDa was seen to be inversely correlated with the amount of pN15 used for reconstitution (Fig. S3), indicating that cross-linking between PsbE and the small subunit of ~4 kDa would be concomitantly inhibited by pN15. It was inferred that this cross-linking partner of 4 kDa is most likely to be the PsbF protein, the partner of PsbE; together PsbE and PsbF form the complete Cyt *b*_559_ entity^6^,^37^. Hence immunoblotting analysis was performed using antibodies raised against PsbF (Fig. 3B). Indeed, a band at approximately 4 kDa was identified as that of PsbF. Of interest, this band disappeared when PSII was treated with EDC in the presence of pN15. This suggested that when pN15 is reconstituted with PSII, PsbF underwent modifications by EDC to such an extent it was no longer recognized by peptide antibodies raised against PsbF. Consistent with this, the cross-linked product, expected to be approximately 13 kDa, was also not detected in the immunoblots. The PsbF antibody used recognised the N-terminal regions of PsbF, those that bind the redox haem on the stromal side (Figs S4 snd S5). Therefore, pN15 would affect the structure around the redox haem of Cyt *b*_559_ in a manner that traverses the membrane.

### pN15 peptide decreases the oxygen-evolving activity of salt-washed PSII membranes

**Figure 4:**
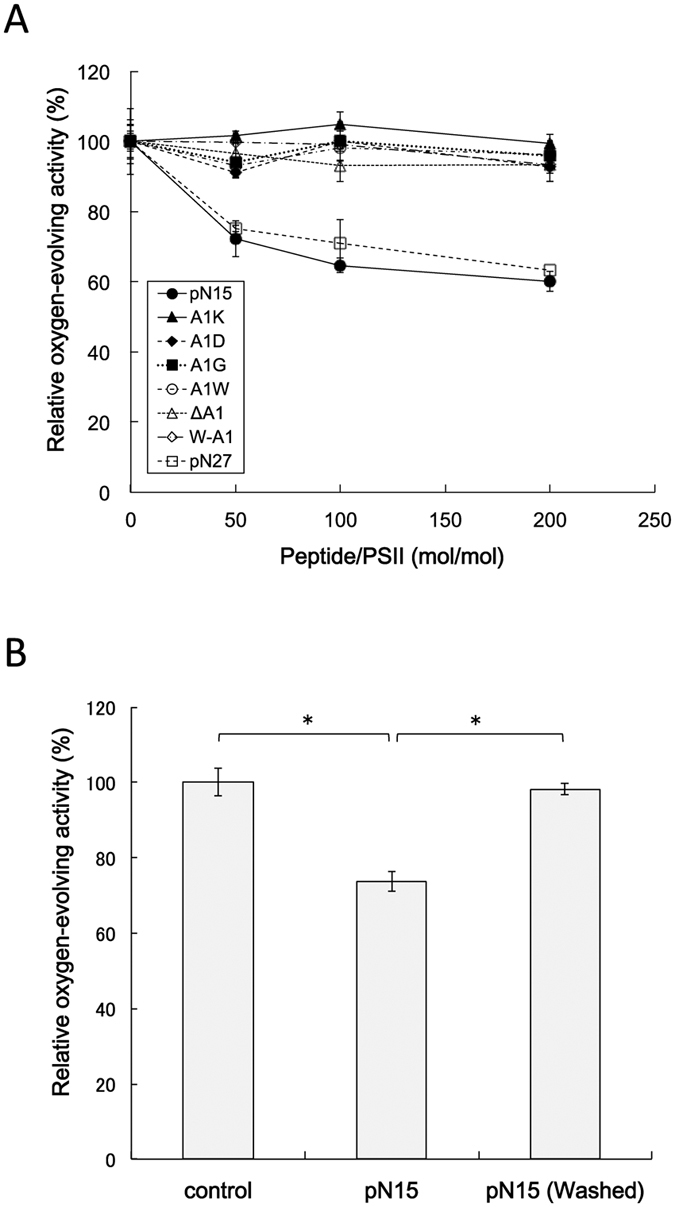
Oxygen-evolving activity of PSII membranes reconstituted with pN15, the N-terminal peptide of PsbP. (**A**) Reconstitution of various pN15 fragments was performed at a molar pN15:PSII ratio of 50:1 to 200:1. PSII oxygen-evolving activity was measured in the presence of 5 mM CaCl_2_ and 5 mM NaCl, and the rate of oxygen-evolution of PSII membranes without reconstitution was set at 100% (251 ~ 317 μmol O_2_ mg Chl^−1^ h^−1^ in independent experiments); *n* = 3, error bars = SEM. (**B**) Oxygen-evolving activity of pN15-reconstituted PSII membranes with or without a washing step. Reconstitution was performed at a molar pN15:PSII ratio of 200:1. Oxygen-evolving activity here was measured in the presence of 5 mM CaCl_2_ and 5 mM NaCl, and the rate of oxygen-evolution for the PSII membranes without reconstitution (the control) was set at 100% (261 μmol O_2_ mg Chl^−1^ h^−1^). The asterisks indicate the significant difference (*p < 0.01, Student’s *t*-test); *n* = 3, error bars = SEM.

To further investigate how the PsbP N-terminus affects the structure and function of PSII, the pN15 peptide fragment was introduced using reconstitution studies and its effect on the water-splitting reaction analysed. The oxygen-evolving capability of reconstituted PSII membranes was measured in the presence of 5 mM CaCl_2_, where reconstitution of PsbP has been shown to be unnecessary for oxygen evolution^38^,^39^ (Fig. S6). Interestingly, the rate of oxygen-evolution for PSII membranes was decreased by reconstitution of pN15 even in the presence of 5 mM CaCl_2_, and the reduction in rate of oxygen-evolution was dependent on the amount of pN15 reconstituted (Fig. 4A). This demonstrates that pN15 has an inhibitory effect on the rate of oxygen evolution. Simultaneous reconstitution of pN15 and full-length (intact) PsbP did not inhibit the oxygen-evolving reaction (Fig. S6). This suggests that intact PsbP can eliminate the inhibitory effect of pN15. Therefore, the inhibitory effect of pN15 was not due to non-specific interaction with PSII.

The effects of pN15-∆A^1^ and other mutated pN15 peptide fragments, in which the N-terminal Ala^1^ was substituted with other residues (Gly, Asp, Lys, or Trp), or an additional Trp residue was added to the N-terminus (depicted as A^1^G, A^1^D, A^1^K, A^1^W, and W-A^1^, respectively), were also investigated. In contrast to the native pN15 fragment, none of these mutated pN15 fragments showed any inhibitory effect on oxygen evolution, indicating that the N-terminal Ala^1^ is crucial for the inhibitory effect of pN15. Furthermore, a pN27 peptide fragment, consisting of the first 27 residues of the N-terminus, was prepared and its reconstitution was observed to decrease the rate of oxygen-evolution by the PSII membranes in a manner similar to pN15. This showed that an extension of peptide length, from 15 up to 27 residues, does not significantly change the inhibitory effect of pN15 on water-splitting. The estimated dissociation constant (K_d_ value) of pN15 to PSII complex was ~5.9 × 10^−7^ M. Such a high K_d_ value certainly suggests that the binding affinity of pN15 to the PSII core complex is relatively low.

Cross-linking experiments using mutated pN15 and pN27 peptide fragments were also performed (Fig. S7). When mutated pN15 peptides were used, the cross-linked band of ~11 kDa was greatly decreased, suggesting that the N-terminal residue of pN15 is indeed important for the interaction with PsbE. Furthermore, when pN27 peptide fragments were used for cross-linking experiments, the new band appeared at ~13 kDa in the stead of the band ~11 kDa. In summary, pN15 interacts with PsbE in the same way as intact PsbP and affects the conformation and interaction between the Cyt *b*_559_ subunits, PsbE and PsbF.

We next examined if the inhibitory effect of pN15 on PSII was reversible or not. The PSII sample reconstituted by pN15 was washed once with the buffer used for reconstitution, and then the oxygen-evolving activity was measured in the presence of 5 mM CaCl_2_ and 5 mM NaCl (Fig. 4B). The rate of oxygen-evolution of PSII, washed after pN15 reconstitution, was restored to almost the same level of the control PSII sample, indicating that the inhibitory effect of pN15 is reversible. It also suggests that the imperfect inhibition of oxygen-evolving activity by pN15 might also be caused by its partial dissociation away from PSII upon dilution with the assay buffer.

### pN15 inhibits the S_1_ to S_2_ transition of the OEC

**Figure 5:**
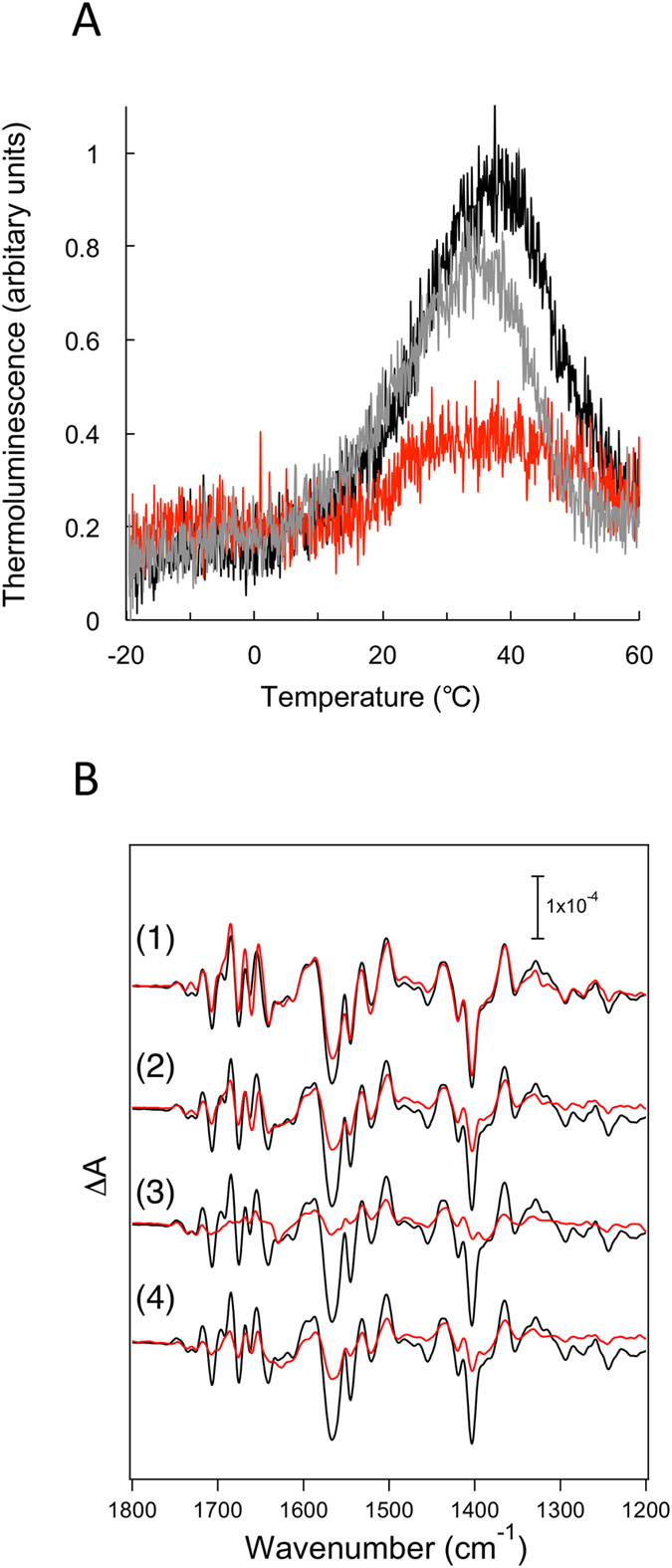
The pN15 peptide inhibits the S_1_ to S_2_ transition of the OEC in PSII. (**A**) Thermoluminescence glow curves of the S_2_/S_3_Q_B_^-^ charge recombination in NaCl-washed (*black line*), pN15-reconstituted (*red line*) and pN15-∆A^1^-reconstituted (*grey line*) PSII membranes. (**B**) The S_2_/S_1_ FTIR difference spectra of NaCl-washed (*black line*) and pN15- or pN15-∆A^1^-reconstituted (*red line*) PSII membranes. PSII membranes were reconstituted with pN15 at a molar pN15:PSII ratio of 50:1 (1), 100:1 (2), and 200:1 (3) and with pN15-∆A^1^ at 200:1 (4).

To examine further the inhibitory effect of pN15 on light-induced charge separation within PSII, thermoluminescence (TL) measurements were conducted on NaCl-washed PSII samples (the control PSII) and pN15-reconstituted PSII membranes (reconstitution was performed at a molar ratio of 200:1) in the presence of 5 mM CaCl_2_ and 5 mM NaCl (Fig. 5A). TL originates from a PSII reaction centre that is re-excited by a charge recombination due to an increase in the temperature within the samples, where light-induced charge pairs in PSII had been freeze-trapped^40^. The B-band arises from a recombination of the S_2_/S_3_ state, of the Mn_4_CaO_5_ cluster, with Q_B_^− ^41. In the control PSII, B-bands were observed around 37 °C, however, B-band intensity in pN15-reconstituted PSII was significantly decreased. Of note, the opposite occurred with pN15-∆A^1^ moderately decreasing the intensity of the B-band. Afterwards, the intensity of the B-band for pN15-reconstituted PSII was restored to that of control PSII sample levels by washing with buffer, suggesting that pN15 reversibly inhibits the S_2_/S_3_Q_B_^-^ charge separation within PSII (Fig. S8).

The inhibitory effect of pN15 on the oxygen-evolving mechanism was further examined by FTIR analysis. FTIR difference spectroscopy is able to detect subtle structural changes coupled to oxygen-evolution, including the conformational changes in polypeptide subunit main chains, amino acid side chains, the core structure of the Mn_4_CaO_5_ cluster, and substrate and functional water molecules^42^. The S_2_/S_1_ FTIR difference spectra of NaCl-washed, and pN15-reconstituted PSII membranes, are shown in Fig. 5B. Prominent bands at 1700–1600 and 1450–1300 cm^−1^ mainly arise from the amide I vibrations (C=O stretches of backbone amides) of polypeptide main chains and the symmetric COO^–^ stretching vibrations of surrounding carboxylate groups, respectively, while bands at 1600–1500 cm^−1^ arise from either the amide II vibrations (NH bends coupled with the CN stretches of backbone amides) or the asymmetric COO^–^ vibrations. It was previously shown that features in the amide I region were perturbed by washing with NaCl but recovered by PsbP binding^20^. However, rebinding of pN15 to NaCl-washed PSII membranes did not recover the amide I bands; on the contrary, it diminished the entire spectral changes in the 1800–1200 cm^−1^ region and the spectral intensity was mostly lost when reconstitution was performed with a molar ratio of 200:1 (pN15:PSII). In contrast, addition of the pN15-∆A^1^ with a ratio of 200:1 showed a moderate inhibitory effect. These data once more indicate that the S_1_ to S_2_ transition was severely inhibited by pN15. To be noted is that differences in the extent of inhibition among the different analyses might be caused by a low binding affinity of pN15 with PSII: Reconstituted PSII membranes were used without dilution in TL analysis, and they were further concentrated as a hydrated film used in FTIR, while samples are necessarily diluted in the measurements of oxygen-evolving activity. Overall, it is concluded that pN15 reversibly inhibits the S_1_ to S_2_ transition of the OEC in PSII.

## Discussion

It is recognized that PsbP induces conformational change around the OEC to allow for Ca^2+^ and Cl^–^ ions to bind with high affinity^20^. In our study, a distinct regulatory mechanism via the N-terminal domain of PsbP has been elucidated, given that the reconstitution of PsbP, as well as pN15, affects the redox potential of Cyt *b*_559_: both convert the Cyt *b*_559_ in NaCl-washed PSII membranes into the HP form, albeit imperfectly. In fact, pN15 interacts with PsbE and affects the structural conformation of Cyt *b*_559_ directly. Interestingly, for the oxygen-evolving activity, FTIR, and TL measurements suggest that pN15 reversibly inhibits the S_1_ to S_2_ transition of the OEC. Currently, a direct relationship between the two observations above has not been demonstrated. One possibility is that some of the oxidative hole around P680 were transferred to the HP form of Cyt *b*_559_, causing a reduction in oxygen-evolution during the presence of pN15. For intact PsbP, its N-terminal sequence did not prevent oxygen-evolution because the secondary electron transfer pathway, via Cyt *b*_559_, is unable to compete with the electron donation from the Mn_4_CaO_5_ cluster in intact PSII^6^. We thus propose that PsbP has a dual function to activate primary electron transfer from the Mn_4_CaO_5_ cluster and also to secure secondary electron transfer to P680^•+^. This would allow a fine balance between the donor and acceptor reactions within PSII to be effected. Our results may also be relevant to previous observations showing that any removal of PsbP and PsbQ affect the electron transfer on the reducing side of PSII^43^,^44^.

Structural differences among different redox forms of Cyt *b*_559_ are unknown. A conversion from the HP to LP, or IP, forms of Cyt *b*_559_ has been observed under various conditions including salt- and Tris-washing of PSII membranes, while the conversion into HP Cyt *b*_559_ has proven to be more difficult to achieve experimentally^6^. It has been proposed that differences in redox potential are due to the protein environment around the haem^7^. We were unable to identify any cross-linking sites between PsbE and PsbF, however, a possible crosslinking site might be found on the stromal side, where the PsbF antibody also recognized its epitope (Fig. S4). In the cyanobacterial PSII structure, Glu^6^ of PsbE and Arg^18^ of PsbF, both highly conserved from cyanobacteria to higher plants, are closely located near the haem and its axial His ligands (Fig. S5). Indeed, mutations of the residues on the cytoplasmic side of Cyt *b*_559_ are reported to affect the redox properties of Cyt *b*_559_^45^. Therefore, it is likely that the interaction of pN15 with PsbE on the lumenal side would change the interaction between PsbE and PsbF in their stromal-facing regions, thus transforming the redox properties of Cyt *b*_559_.

It has been proposed that PsbP has a “catalytic” function, in addition to its structural role as an OEC subunit protecting the Mn_4_CaO_5_ cluster during the assembly of PSII^15^,^16^. In fact, complete elimination of PsbP in an *Arabidopsis* mutant impairs the photo-autotrophy that causes a seedling-lethal phenotype, while a minimum amount of PsbP enables photoautotrophic growth and subsequent accumulation of the PSII reaction centre^21^,^22^,^23^,^24^. In higher plants, the *de novo* biogenesis of PSII, as well as the repair of photo-damaged PSII, occurs in stroma-exposed thylakoid membranes, while the PSII supercomplex accumulates in the stacked granal regions^46^. It was observed that the LP form of Cyt *b*_559_ is mostly present in the stromal membranes, while the HP form is enriched in the grana^47^. PsbP mainly localizes to the grana, but a significant amount of PsbP is present in a free form, or loosely associated with the thylakoid membranes^48^,^49^. Thus it is likely that PsbP does have a role in fine-tuning internal electron transfer within PSII in order to reduce the lifetime of P680^•+^ during the assembly of the OEC, protecting it from excess energy.

In cyanobacteria, a PsbP homolog, CyanoP, is proposed to function as an assembly factor for PSII^50^,^51^, but its exact function is still unclear^52^. Due to CyanoP not having an N-terminal extension sequence, it is probable that other proteins may have such a function, similar to our observations with PsbP, in cyanobacteria. A recent study proposed that Psb30, absent in green plants, interacts with PsbF on the lumenal side of the thylakoid membrane and affects the redox properties of Cyt *b*_559_ in *Synechococcus elongatus;* this would suggest the interaction on the lumenal side can affect the properties of the haem within Cyt *b*_559_, a haem that is positioned on the stromal side^53^. It is also possible that PsbV, which also has an interaction with PsbE in a manner similar to PsbP in the cyanobacterial crystal structure, may have a role to regulate the internal electron transfer within PSII. Further studies are certainly necessary to elucidate how the different composition and expression of the extrinsic proteins among photo-oxygenic organisms contribute in regulating, and tuning the efficiency of, the internal electron transfer of PSII.

## Materials and Methods

### Preparation of peptide fragments

Peptide fragments were produced by Japan Bio Services (Saitama, Japan). The C-terminus of each peptide fragment was amidated and purity was confirmed to be 95~97% by High-Performance Liquid Chromatography (HPLC). Each peptide powder was dissolved in a MES buffer (25 mM MES-NaOH, pH 6.5) before use.

### Plasmid construction, recombinant protein expression and purification

The recombinant PsbP-WT and –∆9 proteins from *Spinacia oleracea* (GenBank Accession number, CAA29055.1) were expressed in the *Escherichia coli* strain BL21(DE3) and purified as described previously^10^,^54^. The presence of the desired mutation in the recombinant protein was confirmed using MALDI-TOF mass spectrometry (Autoflex III; Bruker Daltonics, MA).

### Reconstitution experiments

Reconstitution of the pN15 peptide fragments and PsbP proteins to NaCl-washed PSII membranes was performed based on a procedure reported previously, with some modification^55^. PSII membranes, isolated from spinach leaves^56^, were treated for 30 min with the buffer containing 1.5 M NaCl on ice to remove PsbP and PsbQ. Then pN15 and PsbP were reconstituted with NaCl-washed PSII using a molecular ratio of 50:1, 100:1, or 200:1 (pN15:PSII) and 4:1 (PsbP:PSII). In the control, MES buffer, without any peptide fragments, was used. After incubation for 1 h on ice, the reconstituted PSII samples were carried forward to determine the state of their redox forms of Cyt *b*_559_, oxygen-evolving activity, FTIR analysis and TL measurements. Where indicated, PSII samples were washed once, before the analysis, with buffer (25 mM MES-NaOH, pH 6.5, 5 mM NaCl, 5 mM CaCl_2_, 0.4 M sucrose). The oxygen-evolving activity of each PSII membrane sample was measured in this same buffer using a Clark-type oxygen-electrode (Hansatech, UK) in the presence of 0.4 mM 2,6-dichloro-*p*-benzoquinone (DCBQ) as an electron acceptor.

### Determination of the redox forms of Cyt *b*_559_

PSII membranes were suspended at a Chl concentration of 75 μg ml^−1^ in buffer (25 mM MES-NaOH, pH 6.5, 5 mM NaCl, 5 mM CaCl_2_, 0.4 M sucrose) and the different redox forms of Cyt *b*_559_ were determined at a wavelength of 559 nm, from the reduced minus oxidized difference absorption spectra between 520 and 580 nm, recorded as described previously^34^,^35^,^57^ using a spectrophotometer equipped with a head-on photomultiplier tube (UV-2600; Shimadzu, Kyoto, Japan). Complete oxidation of Cyt *b*_559_ was achieved by treatment with 2 mM potassium ferricyanide (midpoint redox potential *E*_m_ ~ 430 mV) followed by its stepwise reduction. The reduction of the HP form (*E*_m_ ~ 400 mV), the IP form (*E*_m_ ~ 200 mV), and the LP form (*E*_m_ ~ 50 mV) of Cyt *b*_559_^7^,^58^,^59^,^60^ were performed by adding 4 mM hydroquinone (*E*_m_ ~ 280 mV), 5 mM sodium ascorbate (*E*_m_ ~ 60 mV), and 10 mM sodium dithionite (*E*_m_ ~ −660 mV) in a step-wise manner. The absorption difference at 559 nm, in difference spectra of hydroquinone-reduced minus ferricyanide-oxidized, ascorbate-reduced minus hydroquinone-reduced, and dithionite-reduced minus ascorbate-reduced Cyt *b*_559_, enable the content of HP, IP, and LP form of Cyt *b*_559_, to be deduced, respectively. Baselines were set by drawing a straight line between absorption differences at 540 and 580 nm.

### Cross-linking experiments

Cross-linking was performed as described previously^27^,^28^. The NaCl-washed PSII membranes, at a concentration of 0.5 mg Chl ml^−1^, were cross-linked with pN15 peptide fragments in buffer (25 mM MES-NaOH, pH 6.5, 5 mM NaCl, 5 mM CaCl_2_, 0.4 M sucrose) containing 6.25 mM EDC and 5 mM *N*-hydroxysulfosuccinimide (sulfo-NHS). Samples were incubated for 2 h in darkness and the reaction was terminated by adding ammonium acetate to a final concentration of 0.2 M. The cross-linked PSII membranes were subjected to SDS-PAGE via 18% SDS-polyacrylamide gel, and separated proteins were transferred to polyvinylidene difluoride (PVDF) membranes and immuno-detected with specific antibodies. Rabbit antibodies against PsbE and PsbF were purchased from Agrisera AB, Sweden. Rabbit antibody against PsbO was provided by the late Dr. A. Watanabe of Tokyo University. Rabbit antibody against D1 was prepared by the authors.

### Thermoluminescence measurements

Thermoluminescence was recorded with an apparatus manufactured by PSI (Brno, Czech Republic). For measurements, a disc of filter paper 5 mm in diameter, was soaked with a total of 10 μg Chl for each PSII sample being investigated. Each disc was then incubated for 2 min at 25 °C in darkness, cooled to −20 °C, and illuminated with a short actinic flash (30 μs). Light emission during sample warming was recorded from −20 °C to 60 °C, at a heating rate of 1 °C s^−1^.

### FTIR analysis

FTIR measurements were performed following the method reported previously^20^,^61^ with some modifications. NaCl-washed PSII membranes were suspended (2.5 mg Chl ml^−1^) in a buffer containing 4 mM MES-NaOH, 40 mM sucrose, 5 mM CaCl_2_, and 5 mM NaCl (pH 6.0). An aliquot of the sample (10 μl) was mixed with 1 μl of 20 mM potassium ferricyanide and a desired volume of 5 mM pN15 (1.4, 2.8, and 5.6 μl for the molar ratio of 50, 100, and 200, respectively, of pN15 to PSII), and lightly dried on a CaF_2_ plate (25 × 25 mm) in an oval shape (6 × 9 mm) under an N_2_ gas flow. The resultant sample film was moderately hydrated by sealing the cell using another CaF_2_ plate and a silicone spacer (0.5 mm in thickness) enclosing 2 μl of 40% (v/v) glycerol solution without touching the sample. The sample temperature was adjusted to 10 °C by circulating cold water in a copper holder. Light-induced S_2_-minus-S_1_ difference spectra (S_2_/S_1_ spectra) were recorded using a spectrophotometer (VERTEX 80, Bruker Optics) equipped with an MCT detector (InfraRed D313-L) at 4 cm^−1^ resolution (20, 61). A Ge filter to cut IR light at >2200 cm^−1^ (Andover, 4.50ILP-25) was placed in the IR path in front of the sample to improve the signal-to-noise ratios of spectra as well as to block a He-Ne laser beam from the interferometer. Illumination was provided by a Q-switched Nd:YAG laser (INDI-40-10; 532 nm, ~ 7 ns full width at half-maximum, and ~ 7 mJ pulse^–1^ cm^–2^ at the sample surface; Spectra-Physics, UK). Single-beam spectra were recorded with 100 scans (~50-s accumulation) before and after single-flash illumination to calculate a difference spectrum, and the measurements were repeated 20 times with an interval of 25 min. In the case of pN15-treated samples, measurements with 20 scans were repeated 100 times with an interval of 5 min. This difference in the durations of scans and dark interval is due to faster relaxation of the S_2_ state in the pN15-treated samples (τ ~ 20–30 s) than in the NaCl-washed sample (τ ~ 150 s).

## Additional Information

**How to cite this article**: Nishimura, T. *et al.* The N-terminal sequence of the extrinsic PsbP protein modulates the redox potential of Cyt *b*_559_ in photosystem II. *Sci. Rep.* **6**, 21490; doi: 10.1038/srep21490 (2016).

## Supplementary Material

Supplementary Information
