# Supplementary material for: The N-terminal sequence of the extrinsic PsbP protein modulates the redox potential of Cyt b559 in photosystem II
Source: Sci Rep. 2016 Feb 18;6:21490. doi: 10.1038/srep21490 (PMC4757834; doi:10.1038/srep21490)
Supplement: Supplementary Information [file srep21490-s1.pdf]

Supplemental Information

The N-terminal sequence of PsbP modulates the redox potential of the Cyt b_{559} in photosystem II

Taishi Nishimura[a], Ryo Nagao[b], Takumi Noguchi[b], Jon Nield[c], Fumihiko Sato[a] & Kentaro Ifuku[a, 1]

[a]Graduate School of Biostudies, Kyoto University, Sakyo-ku, Kyoto 606-8502, Japan;
[b]Graduate School of Science, Nagoya University, Aichi 464-8602, Japan
[c]School of Biological and Chemical Sciences, Queen Mary University of London, London E1 4NS, United Kingdom

[1]Corresponding author

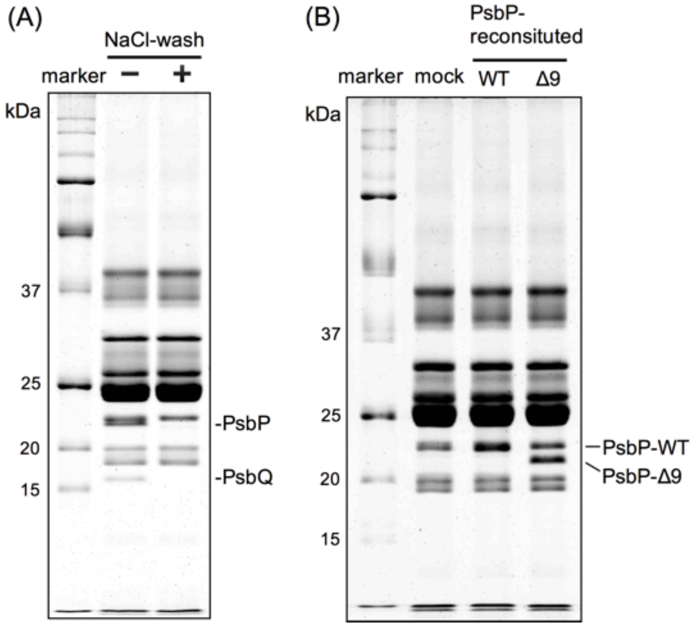

Fig. S1. (A) SDS-PAGE analysis of PSII membranes before (-) and after (+) 1.5 M NaCl washing. The band positions for PsbP and PsbQ are indicated on the right. Each lane of the gel contains proteins corresponding to 5 µg Chl. (B) SDS-PAGE analysis of NaCl-washed PSII reconstituted with the buffer (mock), PsbP-WT, and –Δ9. The band positions for PsbP-WT and –Δ9 are indicated on the right. Each lane of the gel contains proteins corresponding to 5 µg Chl.

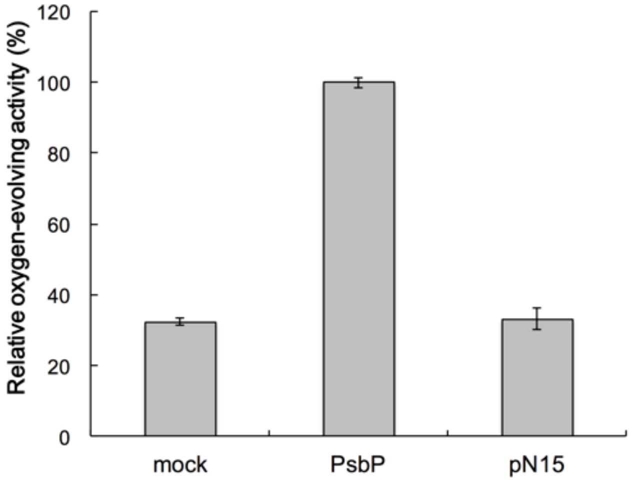

Fig. S2. Oxygen-evolving activity of NaCl-washed PSII (Control) and PSII reconstituted with PsbP-WT and pN15. Oxygen-evolving activity was measured in the absence of Ca^{2+} and Cl^{-} ions. The activity of WT-reconstituted PSII (187 μmol O_2 mg Chl^{-1} hr^{-1}) was set as 100%. *n* = 3, error bars = SEM.

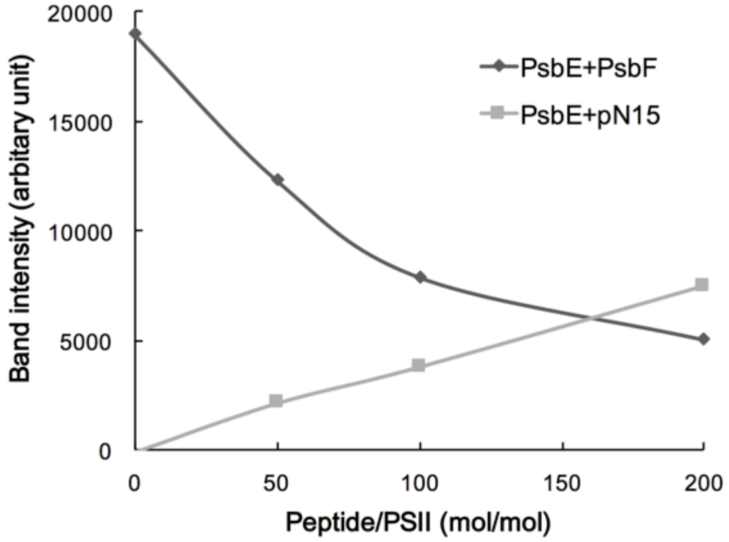

Fig. S3. Quantification of the cross-linked peptides of pN15-PsbE and PsbE-PsbF. The intensities of corresponding bands in the immunoblot (Fig. 3a) were quantified using Image J software.

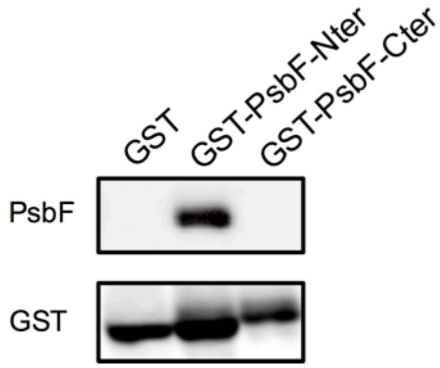

Fig. S4. The antibody against PsbF used in Fig. 2b specificity reacted with N-terminal 14 residues of PsbF. Glutathione S-transferase (GST), GST-fused with N-terminal 14 residues of PsbF (GST-PsbF-Nter), and GST-fused with C-terminal 24 residues of PsbF proteins (GST-PsbF-Cter) were expressed in *Escherichia coli* strain BL21. Disrupted cell suspensions were separated by SDS-PAGE and immunodetected with antibody against PsbF and GST.

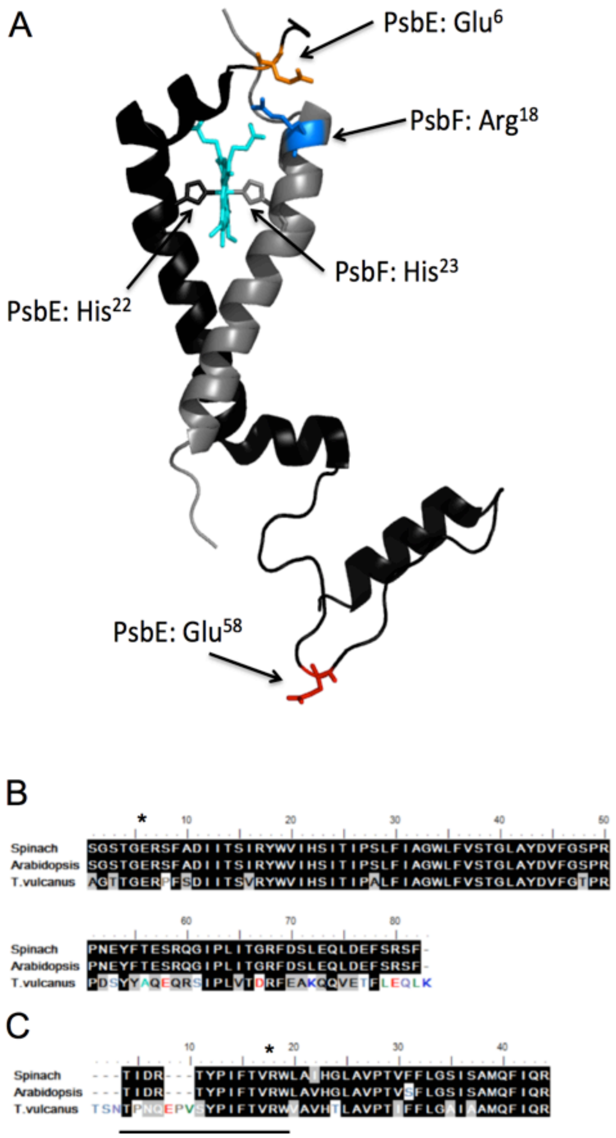

Fig. S5. (A) Structure of Cyt b_{559} in cyanobacterial PSII from the 1.9 Å model of Umena *et al.*[5]. The a subunit (PsbE) and b subunit (PsbF) of Cyt b_{559} are shown as black and gray ribbon model, respectively. Heme (cyan) and its axial ligands (His^{23} of PsbE and His^{22} of PsbF) are shown as stick models. Glu^{6} of PsbE and Arg^{18} of PsbF, which can be crosslinked by EDC, are shown as orange and blue stick models. The Glu^{58}, corresponding to Glu^{57} in spinach PsbE, which interacts with PsbP, is shown as red stick model. (B) Amino acid sequence alignment of PsbE from *Spinacia oleracea*, *Arabidopsis thaliana*, and *Thermosynechococcus vulcanus*. (C) Amino acid sequence alignment of PsbF from *S. oleracea*, *A. thaliana*, and *T. vulcanus*. Glu^{6} of PsbE and Arg^{18} of PsbF are indicated by asterisks. A putative epitope sequence of the PsbF-antibody is underlined.

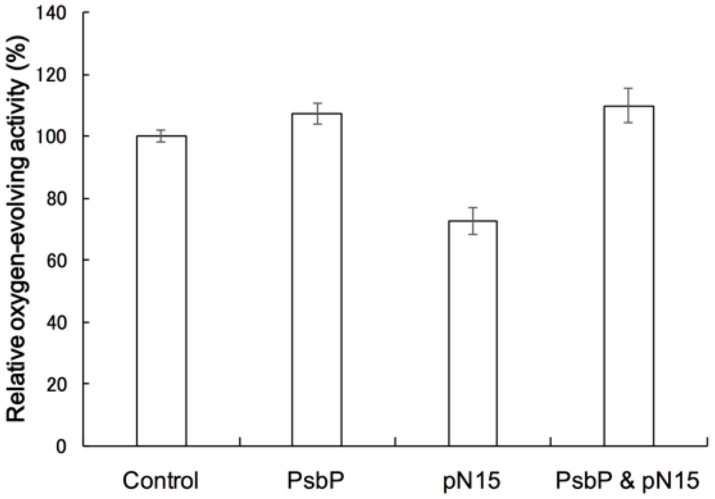

Fig. S6. Oxygen-evolving activity of NaCl-washed PSII (Control) and PSII reconstituted with PsbP-WT and pN15. Oxygen-evolving activity was measured in the presence of 5 mM $CaCl_2$ and 5 mM NaCl, and the rate of oxygen-evolution of PSII membranes without reconsitution (175 μmol O_2 mg Chl^{-1} hr^{-1}) was set as 100%. *n* = 3, error bars = SEM.

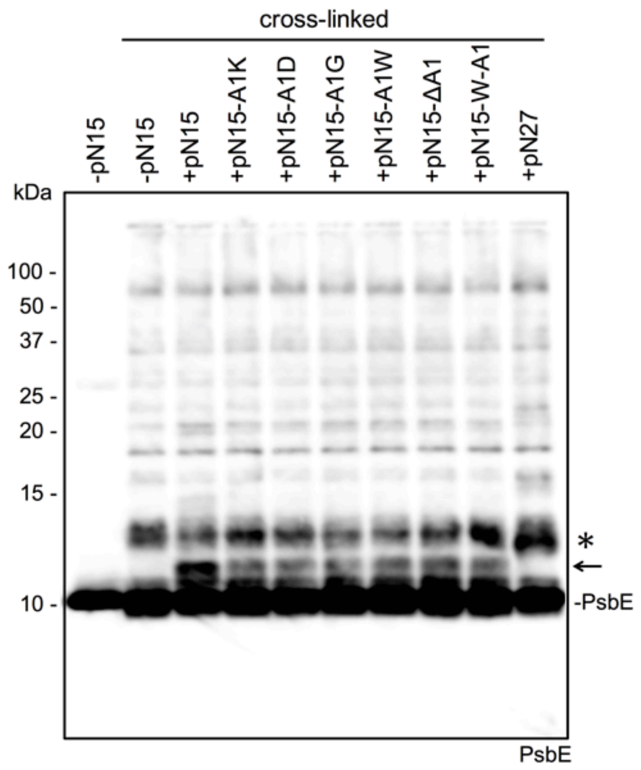

Fig. S7. Cross-linking of mutated pN15 fragments and pN27 fragment with PSII membranes using EDC and sulfo-NHS. NaCl-washed PSII membranes were cross-linked with native or mutated pN15 fragments and pN27 fragment at a molar pN15:PSII ratio of 200:1. Proteins corresponding to 3 µg Chl were loaded onto each lane and detected with antisera against PsbE. Arrows around 11 kDa indicates the cross-linked peptide including pN15 and PsbE. The cross-linked peptide around 14 kDa including pN27 and PsbE is indicated by astarisk. Original position of PsbE (9 kDa) is also indicated.

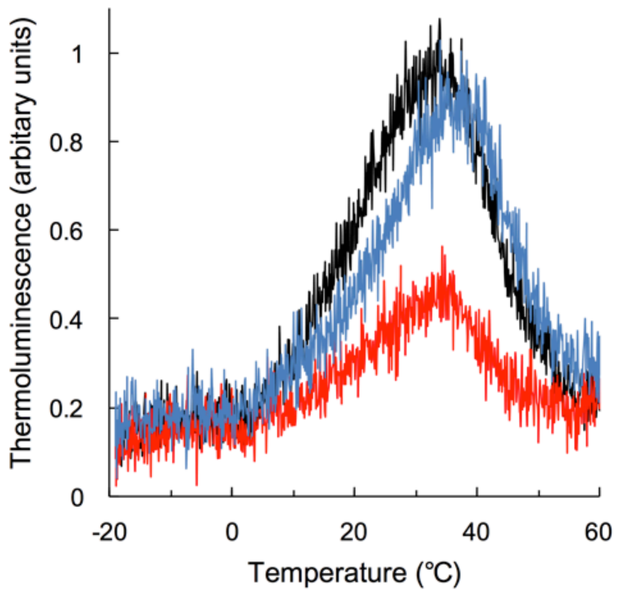

Fig. S8. Thermoluminescence glow curves of the $S_2/S_3Q_B^-$ charge recombination in NaCl-washed (*black line*), pN15-reconstituted (*red line*), and pN15-reconstituted and washed (*blue line*) PSII membranes.
